# Ca^2+^ channel clustering with insulin-containing granules is disturbed in type 2 diabetes

**DOI:** 10.1172/JCI88491

**Published:** 2017-05-08

**Authors:** Nikhil R. Gandasi, Peng Yin, Michela Riz, Margarita V. Chibalina, Giuliana Cortese, Per-Eric Lund, Victor Matveev, Patrik Rorsman, Arthur Sherman, Morten G. Pedersen, Sebastian Barg

**Affiliations:** 1 Medical Cell Biology, Uppsala University, Uppsala, Sweden.; 2 Department of Information Engineering, University of Padova, Padova, Italy.; 3 Oxford Centre for Diabetes, Endocrinology and Metabolism, University of Oxford, Oxford, United Kingdom.; 4 Department of Statistical Sciences, University of Padova, Padova, Italy.; 5 Department of Mathematical Sciences, New Jersey Institute of Technology, Newark, New Jersey, USA.; 6 Laboratory of Biological Modeling, National Institute of Diabetes and Digestive and Kidney Diseases (NIDDK), NIH, Bethesda, Maryland, USA.

## Abstract

Loss of first-phase insulin secretion is an early sign of developing type 2 diabetes (T2D). Ca^2+^ entry through voltage-gated L-type Ca^2+^ channels triggers exocytosis of insulin-containing granules in pancreatic β cells and is required for the postprandial spike in insulin secretion. Using high-resolution microscopy, we have identified a subset of docked insulin granules in human β cells and rat-derived clonal insulin 1 (INS1) cells for which localized Ca^2+^ influx triggers exocytosis with high probability and minimal latency. This immediately releasable pool (IRP) of granules, identified both structurally and functionally, was absent in β cells from human T2D donors and in INS1 cells cultured in fatty acids that mimic the diabetic state. Upon arrival at the plasma membrane, IRP granules slowly associated with 15 to 20 L-type channels. We determined that recruitment depended on a direct interaction with the synaptic protein Munc13, because expression of the II–III loop of the channel, the C2 domain of Munc13-1, or of Munc13-1 with a mutated C2 domain all disrupted L-type channel clustering at granules and ablated fast exocytosis. Thus, rapid insulin secretion requires Munc13-mediated recruitment of L-type Ca^2+^ channels in close proximity to insulin granules. Loss of this organization underlies disturbed insulin secretion kinetics in T2D.

## Introduction

Insulin is the body’s principal hypoglycemic hormone and is released from pancreatic β cells by regulated exocytosis of secretory granules. Glucose elicits β cell electrical activity and Ca^2+^ influx through voltage-gated Ca^2+^ channels, which in turn triggers exocytosis (1). Genetic ablation of L-type Ca^2+^ channels in mouse β cells prevents rapid exocytosis of insulin granules and is associated with deficient insulin secretion (2) reminiscent of human type 2 diabetes (T2D) (3). Although β cells contain relatively few L-type Ca^2+^ channels (500/cell) (4), a limited pool of granules can be released with latencies as short as 5 to 10 ms. Exocytosis in β cells requires relatively high [Ca^2+^] (*K_D_* ~20 μM), while bulk cytosolic [Ca^2+^] remains below 1 μM during glucose stimulation (4–7). These granules are insensitive to cytosolic Ca^2+^ buffering, suggesting that they are situated near Ca^2+^ influx sites (4, 8, 9). According to this concept of “positional priming,” granules near voltage-gated Ca^2+^ channels experience localized Ca^2+^ changes that are faster, more transient, and much larger than those in the average cytosol, resulting in exocytosis that is well synchronized with Ca^2+^ channel opening (10–12). Indeed, short depolarizations elicit microdomains of elevated Ca^2+^ in mouse β cells (9), and the rapid kinetics of exocytosis in human β cells suggest the existence of a limited pool of granules located at L-type Ca^2+^ channels (13, 14).

The majority of Ca^2+^ entry into human and rodent β cells occurs via L- and P/Q-type Ca^2+^ channels (reviewed in ref. 15). Mouse β cells express the L_C_-type channel (CaV1.2) (4, 16), while rat and human β cells express L_D_ (CaV1.3) (17–19). In humans, both isoforms are likely important for insulin secretion (17, 20). L- and P/Q-type channels bind to proteins of the exocytosis machinery, such as syntaxin, synaptotagmin, and active zone proteins such as Rab3-interacting molecule (RIM) and Munc13 (21, 22), which can alter the channels’ gating properties (23–27). The interaction involves a region located in the cytosolic loop between transmembrane domains II and III, corresponding to the synaptic protein interaction (synprint) site in neuronal Ca^2+^ channels (28). A similar peptide derived from the II–III loop of the L_C_ channel (CaV1.2) selectively ablates fast exocytosis in mouse β cells (4, 29–31). The active zone proteins Munc13 and RIM bind to the synprint site via their C2 domain and orchestrate the clustering of Ca^2+^ channels in neuronal synapses (21, 22). Although β cells lack ultrastructurally identifiable active zones, they express a number of active zone proteins, including Munc13 and RIM2 (32), that could direct exocytosis to certain areas in the cell (33) or help organize individual release sites.

Here, we used high-resolution live-cell imaging to directly assess the spatial relation between granules and Ca^2+^ channels in human β cells and the insulin-secreting cell line, INS-1. We show that L-type Ca^2+^ channels are recruited to a subset of the docked granules, probably by direct interaction with Munc13 at the release site. Functionally, this places microdomains of tens of μM Ca^2+^ near certain granules, resulting in a rapid exocytosis that is synchronized with the depolarization, while global Ca^2+^ is less important. Intriguingly, this organization is absent in β cells from human T2D donors, suggesting a molecular mechanism for the early loss of first-phase secretion in the disease.

## Results

### Localized Ca^2+^ entry into human β cells.

**Figure 1:**
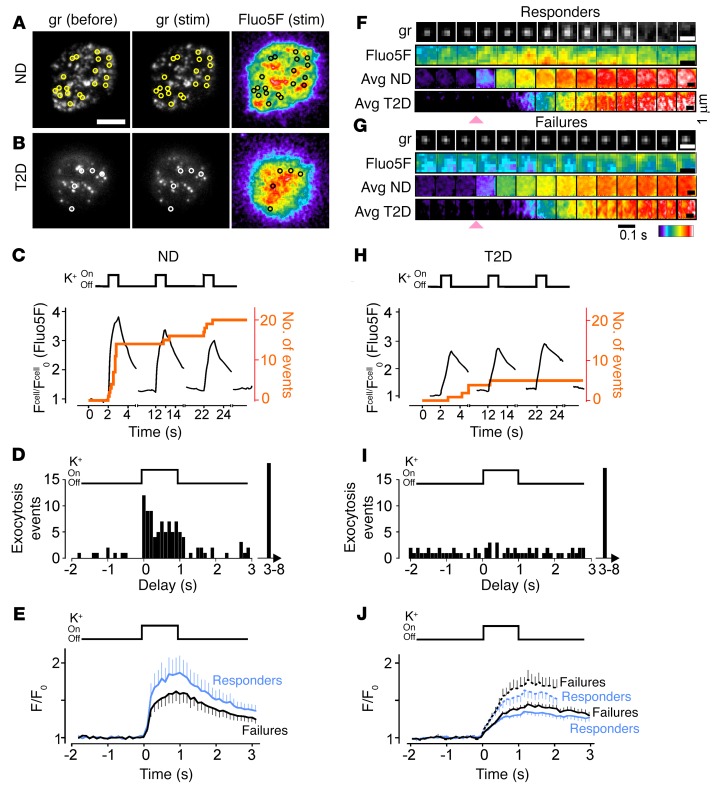
Local calcium influx at exocytosing granules in human islet cells. (**A** and **B**) TIRF images of a ND (**A**) and T2D (**B**) human β cell expressing NPY-mCherry (see “gr,” granule) and loaded with the Ca^2+^ sensor Fluo5F (right), before and during (stim) stimulation with 75 mM K^+^. Circles indicate the location of exocytosis events. Scale bar: 5 μm (**A** and **B**). (**C**) Time course of Fluo5F-Ca^2+^ fluorescence (the whole-cell signal was normalized to prestimulation, F^cell^/F^cell^_0_; black lines) and cumulative exocytosis events (orange line) in a cell periodically stimulated with K^+^ as indicated. The stimulation was carried out for 1 second, with an interval of 9 seconds. Imaging was performed from 2 seconds before until 2 seconds after each pulse. (**D**) Exocytosis events as a function of time relative to the most recent K^+^ pulse in 22 cells, as in **A**. (**E**) Average Fluo5F fluorescence from responders (blue) and failures (black), aligned to the onset of the K^+^ application and the center position of each granule. There were 102 granules each in 22 cells from 8 ND donors. *P* = 0.033, by Student’s *t* test, for the difference in peak amplitude. (**F**) Examples of an individual granule undergoing exocytosis (gr), the Fluo5F signal for the same granule (Fluo5F), the average Fluo5F signals for 102 responders in ND cells (Avg ND), and 104 responders in T2D cells (Avg T2D). Sequences were aligned to the onset of K^+^ application (red arrow). Note the different spatial scale for the example and the averages. (**G**) As in **F**, but for granules that failed to undergo exocytosis (failures). In **F** and **G**, the image frames are shown for every 0.1 second. Arrowheads indicate onset of stimulation. Scale bars: 1 μm (**F** and **G**). (**H**–**J**) As in **C**–**E**, but for 104 granules each in 26 cells from 7 T2D donors. Dashed lines in **J** indicate a subset of 8 ND cells with the highest cell-averaged Fluo5F/Ca^2+^ responses.

We simultaneously imaged submembrane [Ca^2+^] and exocytosis in β cells from nondiabetic (ND) human donors (Supplemental Figure 9; supplemental material available online with this article; https://doi.org/10.1172/JCI88491DS1 for details) using total internal reflection fluorescence (TIRF) microscopy (Figure 1, A and B). The cells expressed neuropeptide Y–mCherry (NPY-mCherry) as a secretable granule marker and were loaded with the fast Ca^2+^ indicator Fluo5F (*K_D_* ~2.3 μM) and the slow Ca^2+^ chelator EGTA (both supplied as acetomethoxy esters). The latter narrows the Fluo5F/Ca^2+^ signal from individual Ca^2+^ influx sites by restricting Ca^2+^ diffusion (34) but does not affect β cell electrical activity or glucose-stimulated insulin secretion (8). The cells were then subjected to pulses of elevated K^+^ (75 mM for 1 s every 10 s) from a pressurized glass pipette. Relatively high K^+^, together with the ATP-sensitive potassium channel (KATP channel) opener diazoxide, essentially clamps the membrane potential, resulting in steep depolarizations and rapid opening of voltage-gated Ca^2+^ channels. During K^+^ pulses, the Fluo5F signal increased by about 4-fold and returned toward baseline in the interval between (Figure 1C, black lines). Small areas of locally high Fluo5F fluorescence could be discerned (Figure 1A), suggesting an uneven distribution of voltage-gated Ca^2+^ entry. Exocytosis was triggered by the depolarizations (Figure 1F, gr) and continued during the 9-second intervals between pulses, in agreement with data from capacitance recordings (17). On average, 0.085 ± 0.010 granules/μm^2^ underwent exocytosis in response to the train of depolarizations (*n* = 120 granules, 22 cells, normalized to the footprint area). Exocytosis was significantly faster during the short depolarizations than during the interval between (63 of 120 events; *P* < 0.0001 by logistic modeling) (Figure 1D).

To understand the spatial relationship of exocytosis and Ca^2+^ influx, we compared the Fluo5F signal at granules that responded to the depolarization with exocytosis (responders) with that at granules that remained docked during the experiment (failures). The individual Fluo5F image sequences suggested local influx of Ca^2+^ near responders (Figure 1, F and G, Fluo5f). This became more obvious when the image sequences were averaged for all responders (Figure 1F). In contrast, at failures, the signal increased gradually and was spatially more uniform (Figure 1G, Avg ND). On average, the peak of the local Fluo5F signal, normalized to its prestimulatory value (F/F_0_), was higher at responder granules than at failures (F/F_0_ = 1.80 ± 0.20 vs. 1.57 ± 0.12 in 102 granules from 22 cells, *P* = 0.033) (Figure 1E). The difference was even greater when we compared granules undergoing exocytosis during a pulse (F/F_0_ = 2.78 ± 0.69, *n* = 19, *P* = 0.002 vs. failures) with those between pulses (F/F_0_ = 1.14 ± 0.06, *n* = 23, *P* = 0.028 vs. during pulses). Thus, depolarization-induced [Ca^2+^] entry occurs preferentially near granules that are released with short latency and high probability. We reached similar conclusions with human β cells stimulated with elevated glucose (Supplemental Figure 1) or tolbutamide (Supplemental Figure 7).

### Localized Ca^2+^ influx is absent in β cells from human diabetic donors.

During the course of this study, we had access to islets from 7 donors with clinically diagnosed T2D (glycated hemoglobin [HbA1c] values between 6.1% and 7.9 %). When these β cells were depolarized with K^+^ (Figure 1, B and H), the Ca^2+^ responses were on average smaller than those in β cells from ND donors (peak F/F_0_= 0.92 ± 0.40 in 26 T2D cells vs. 1.80 ± 0.20 in 22 ND cells, *P* = 0.05) (Figure 1, H and J). Inspection of the traces revealed that this was due to a large fraction of cells with very small Ca^2+^ responses in the T2D group. Exocytosis occurred with slight preference during the 1‑second depolarizations and continued during the 9‑second interval between (24 vs. 97 events, *P* = 0.0004 by logistic modeling) (Figure 1I). However, the estimated probability per time unit for exocytosis to occur during the pulse, rather than between stimuli, was significantly lower in T2D cells than in ND cells (*P* = 0.0001 by logistic modeling). Notably, Ca^2+^ influx in T2D β cells was not localized to granules (Figure 1G, Avg T2D), and the peak Fluo5F/Ca^2+^ signal in responders was not higher than that in failures (0.92 ± 0.40 vs. 1.38 ± 0.09, *n* = 102–105 granules in 26 vs. 22 cells), even in the few cells that had normal cell-averaged Ca^2+^ amplitudes (Figure 1J, dotted lines). We conclude that both granule-localized Ca^2+^ influx and the synchronization between depolarization and exocytosis are disturbed in T2D β cells.

### Localized Ca^2+^ entry into INS1 cells.

**Figure 2:**
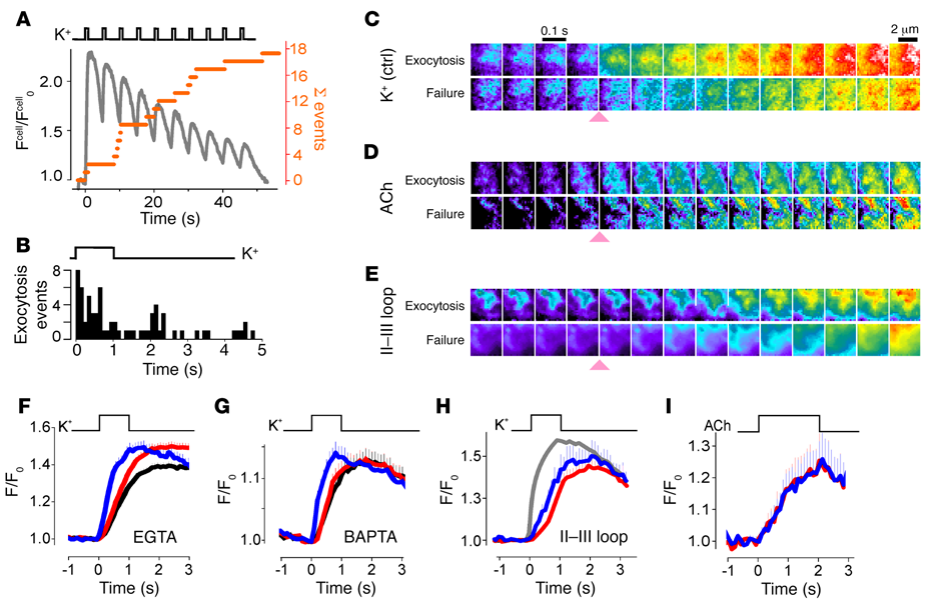
Local calcium influx at exocytosing granules in INS1 cells. (**A**) Cumulative exocytosis events (orange) and cell-averaged lyn-R-GECO-Ca^2+^ fluorescence (F^cell^/F^cell^_0_, black) in an INS1 cell periodically stimulated with K^+^, as indicated. See Supplemental Figure 2 for cell images. (**B**) Frequency of exocytosis events in 15 cells as in **A**, relative to the most recent K^+^ pulse. (**C**) Average images of lyn-R-GECO fluorescence centered on granules undergoing exocytosis or not (Failure) and temporally aligned to the onset of application of 75 mM K^+^ (pink arrowhead); 68 granules each in 15 cells. (**D**) As in **C**, but for cells stimulated with 250 μM ACh (24 granules each in 10 cells). (**E**) As in **C**, but for cells expressing the II–III loop fragment and stimulated with 75 mM K^+^ (30 granules each in 9 cells). Arrowheads indicate onset of stimulation. Scale bar: 2 μm (**C**–**E**). (**F**) Average lyn-R-GECO-Ca^2+^ fluorescence at granules (F/F_0_) undergoing exocytosis (responders, blue), failures (red), and random locations (black) during the first K^+^ pulse. The cells were loaded with EGTA-AM, and 75 mM K^+^ was applied as indicated (12 cells with 67 responders and 200 failures). (**G**) As in **F**, but for cells preloaded with BAPTA-AM (14 cells, 43 granules each). (**H**) As in **F**, but for cells expressing the II–III loop fragment and stimulated for 2 seconds (9 cells, 30 granules each). For comparison, the signal at responders in untransfected cells is shown in gray (8 cells, 45 granules). (**I**) As in **F**, but for cells stimulated with ACh for 2 seconds (10 cells, 24 granules each).

We also observed granule-localized Ca^2+^ influx into INS1-832/13 cells, a widely used insulin-secreting rodent cell line that shares many features with primary β cells and is easy to transfect (35). We imaged submembrane [Ca^2+^] with a membrane-targeted version of R-GECO (lyn-R-GECO) in EGTA-loaded cells. As in the human cells, short, repeated pulses of elevated K^+^ (1 s every 5 s) or trains of voltage-clamp depolarizations resulted in pulsatile increases in submembrane [Ca^2+^] (Figure 2A and Supplemental Figure 2) and elicited partially synchronized exocytosis of NPY-mCherry–labeled granules (Figure 2, A and B). Exocytosis was significantly faster during the K^+^ pulses than in the intervals between stimuli (36 events vs. 11, *P* < 0.0001 by χ^2^ test) (Figure 2B). Spatiotemporal averaging of the GECO images revealed localized Ca^2+^ influx at responder granules, but not at failures (Figure 2C), which corresponded with faster GECO/Ca^2+^ rise times at responders compared with failures (*t_1/2_*= 0.37 ± 0.02 s vs. 0.51 ± 0.03 s, *P* = 0.00012, by Wilcoxon Mann-Whitney *U* test, *n* = 68 responders vs. 200 failures in 12 cells) (Figure 2F). For granules undergoing exocytosis during the first depolarization, the rise times were even shorter (*t_1/2_* = 0.32 ± 0.08 s, *n* = 8 granules). In cells loaded with the faster Ca^2+^ chelator BAPTA [1,2-bis(*o*-aminophenoxy)ethane-*N*,*N*,*N*′,*N*′-tetraacetic acid], [Ca^2+^] still rose faster at responders than at failures but was slowed in both groups relative to EGTA-loaded cells (Figure 2G). When the cells were instead stimulated with short pulses of acetylcholine (ACh) (250 μM) to release Ca^2+^ from intracellular stores, the GECO/Ca^2+^ signal was no longer localized to granules (Figure 2, D and I, and Supplemental Figure 1A). Likewise, expression of the II–III loop fragment of CaV1.2 (amino acids 726–985), which interferes with binding of CaV1.2 to synaptotagmin (30), prevented granule-localized Ca^2+^ influx (Figure 2, E and H). In both cases, the rise in [Ca^2+^] at responders was slower compared with the control but was similar to that at failures.

### Release probability varies with the granules’ proximity to Ca^2+^ influx sites.

**Figure 3:**
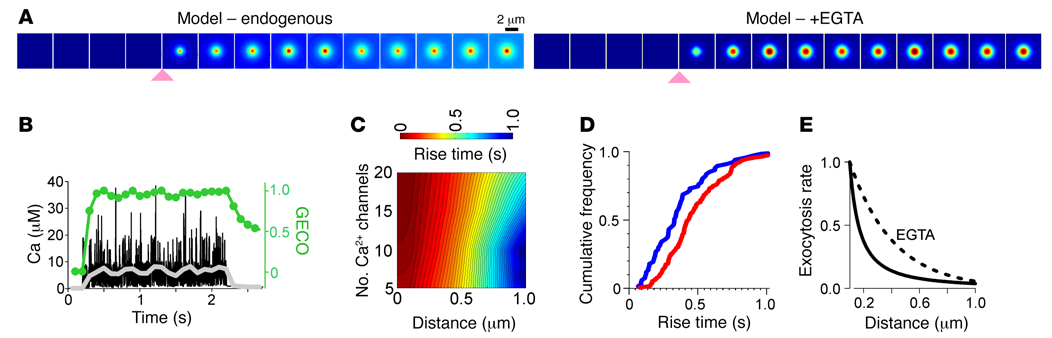
Modeling of Ca^2+^ influx. (**A**) Modeled GECO/Ca^2+^ signal, assuming 15 L-type channels in the center and either endogenous buffering or added EGTA (1 mM). Arrowheads indicate the onset of stimulation. Image frames are shown for every 0.1 second. Scale bar: 2 μm. (**B**) Modeled time course of the [Ca^2+^] (black) and GECO signal (green) in a circle with a diameter of 75 nm and centered on a cluster of 15 Ca^2+^ channels, assuming no added EGTA. The [Ca^2+^] average over 0.1 second time intervals is shown in gray. (**C**) Theoretical GECO rise times (color coded) as a function of the Ca^2+^ channel number in the cluster and the distance from the cluster’s center. (**D**) Cumulative histograms of GECO rise times for responders (blue) and failures (red) for the experiments depicted in Figure 2G (*P* = 0.00012, by Wilcoxon Mann-Whitney *U* test). (**E**) Exocytosis probability, normalized to the probability at *d* = 0.1 μm, as a function of the distance to the Ca^2+^ channel cluster; based on data in Figure 2, C and F, and Supplemental Figure 3 and time-to-event statistics and assuming no added buffering (solid line) or 1 mM EGTA (dotted line).

We mathematically modeled Ca^2+^ influx through clusters of 3 to 20 Ca^2+^ channels with realistic opening kinetics in space and time (see Methods), assuming either added cytosolic EGTA (1 mM) or no exogenous Ca^2+^ buffer. This analytical approach indicated that microdomains with time-averaged [Ca^2+^] of greater than 5 μM and a radius similar to that of a granule (100 nm) form around Ca^2+^ channels (Figure 3, A and B, gray lines, and Supplemental Figure 3E). Within the microdomains, [Ca^2+^] fluctuates rapidly as a result of stochastic channel gating and reaches peak values of more than 20 μM (Figure 3B, black). The theoretical Ca^2+^ signal was then convolved with the known characteristics of our imaging system and the GECO sensor (Figure 3, A and B, green, and Supplemental Figure 3, A and B), giving us the theoretical relationship among the GECO/Ca^2+^ rise time, the distance to the Ca^2+^ entry site, and the number of clustered channels (Figure 3C). Accordingly, the rise time reflects the distance to the influx site and, to a lesser degree, the number of channels at its center. This allowed us to use the experimental rise times from INS1 cells (Figure 3D, corresponding to Figure 2G) to estimate the distance of a granule from the nearest Ca^2+^ influx site (Supplemental Figure 3, C and D). The estimated distances were inserted into a Cox regression model that treats all exocytosis events in a cell as clustered data (time-to-event statistical analysis [ref. 36]; see Methods). This analysis indicates that the rate of exocytosis drops by approximately 50% when the rise time doubles (hazard ratio [HR] 0.49, 95% CI [0.36, 0.68], *P* < 0.0001). Further, it allowed us to calculate the exocytosis rate (cumulative hazard) as a function of the distance to Ca^2+^ channels (Figure 3E). Accordingly, a granule’s release probability is 5- to 10-fold higher when the Ca^2+^ channel cluster is located at the periphery of the release site, compared with when it is an additional 0.5 μm away.

### L-type Ca^2+^ channels cocluster with Munc13 at a subset of docked granules.

**Figure 4:**
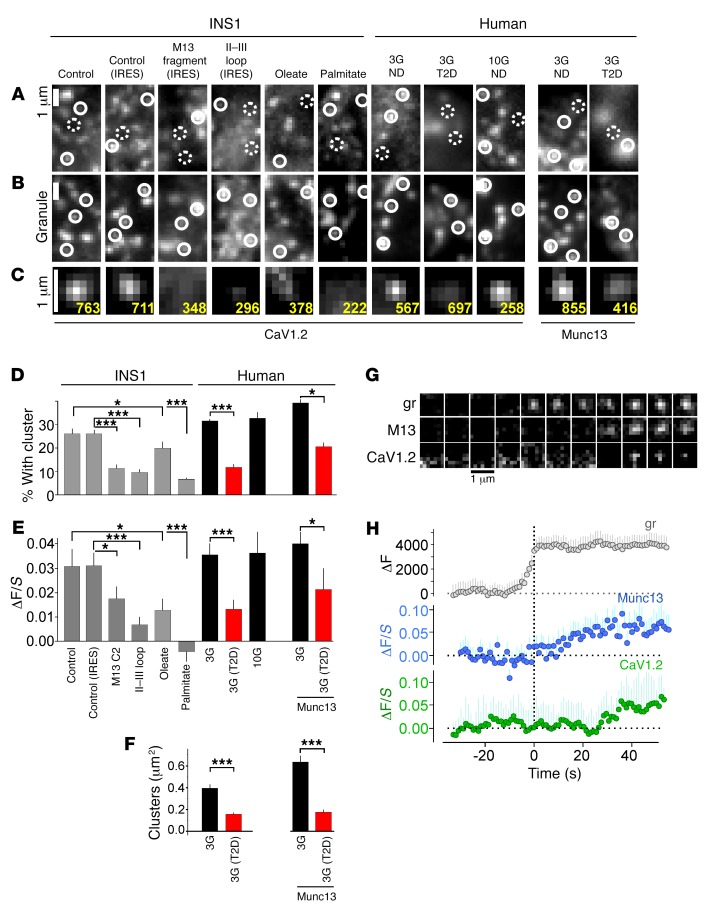
L-type Ca^2+^ channels and Munc13 cluster at docked insulin granules. (**A** and **B**) Images showing parts of INS1 or human β cells coexpressing EGFP-CaV1.2 or Munc13-EGFP as indicated (**A**), together with the granule marker NPY-mCherry (**B**). Solid circles indicate granules with associated CaV1.2/Munc13 clusters, and dotted circles indicate granules without the cluster. Conditions for INS1 (21–52 cells) cells are: control (3 mM glucose); IRES vector control [bicistronic p(empty)IRES-NPY24 mCherry]; overexpression of the Munc13 C2-domain fragment using the IRES vector (M13); the CaV1.2 II–III loop fragment (II–III loop) using the IRES vector; and long-term exposure to 0.5 mM oleate or palmitate. Conditions for human β cells are: 3 or 10 mM glucose (3G, 10G) in ND (20–34 cells, 3 donors) or T2D (31–52 cells, 3 donors) cells. Scale bars: 1 μm. (**C**) Average images of EGFP-CaV1.2 or Munc13-EGFP spatially aligned to the location of docked granules; conditions as in **A** and **B**. The number of analyzed granules is shown in yellow. Scale bar: 1 μm. (**D**–**F**) Quantification of EGFP-CaV1.2 or Munc13-EGFP clusters shown in **A** and **B** as (**D**) the percentage of granules associated with a cluster, (**E**) granule-associated fluorescence (ΔF/*S*), and (**F**) cluster density. The ΔF/*S* for EGFP-CaV1.2 was essentially zero at random locations (–0.004 ± 0.001, 38 cells; *P* < 0.0001, by Student’s *t* test). *P < 0.05 and ***P < 0.001, by Student’s *t* test. (**G**) Example of a granule docking in INS1 cells and corresponding Munc13-EGFP (M13) or EGFP-CaV1.2 signals (separate cells). Scale bar: 1 μm. (**H**) Quantification of granule (gray) and corresponding Munc13-EGFP (blue) or EGFP-CaV1.2 signals (green) aligned to the moment of docking (34 and 21 granules in 12 and 9 cells, respectively).

We expressed the pore-forming α subunit of the L-type Ca^2+^ channel, N-terminally tagged with enhanced GFP (EGFP-CaV1.2), and confirmed that it traffics correctly to the plasma membrane (Supplemental Figure 4A) and forms functional Ca^2+^ channels (Supplemental Figure 4, B–E). TIRF microscopy showed a punctate distribution of EGFP-CaV1.2 in the membrane of both human β cells and INS1 cells (Figure 4A), reminiscent of the pattern obtained earlier by immunostaining in mouse β cells (4). In most cells (83% ± 2% in 416 INS1 cells), the tagged channel formed clusters that were usually diffraction limited in size (<0.2 μm) (Figure 4A). In cells coexpressing the granule marker NPY-mCherry, the vast majority of granules visible in TIRF were docked and immobile at the membrane (37); just over 25% of these docked granules colocalized with a EGFP-CaV1.2 cluster (Figure 4D), compared with 1.1% ± 0.1% at random positions (data not shown). Colocalization was also apparent when we excised small squares from the EGFP-CaV1.2 images, each centered on the location of a randomly chosen granule (>7 per cell), and then averaged all squares (Figure 4C, Control). We quantified the apparent affinity of EGFP-CaV1.2 for granule sites by measuring the local fluorescence specifically associated with granules, normalized for expression level (ΔF/S, see Methods) (38). At the location of docked INS1 cell granules, the ΔF/S was approximately 0.03 (Figure 4D, gray bars). When either the II–III loop fragment or the C2 domain of Munc13-1 was coexpressed, EGFP-CaV1.2 still formed clusters in the plasma membrane (Figure 4A), but its binding to granules (ΔF/S) was strongly reduced (Figure 4, B–E). Likewise, long-term culture with fatty acids, to emulate the diabetogenic action of a high-fat diet (HFD) (39), decreased binding of EGFP-CaV1.2 to insulin granules (Figure 4, C–E). In human ND β cells, EGFP-CaV1.2 localized docked granules to an extent similar to that seen in INS1 cells (Figure 4, A–C), resulting in a ΔF/S of approximately 0.35, regardless of the extracellular glucose concentration (Figure 4E, black bars). In contrast, in cells from T2D donors, only one-tenth of the granules associated with a CaV1.2 cluster. This is likely the consequence of reduced binding of CaV1.2 to granules (Figure 4, D and E, red bars) as well as overall fewer CaV1.2 clusters (Figure 4F). We performed similar experiments with EGFP-tagged Munc13-1 (Munc13-EGFP), because it binds to the synprint domain of voltage-gated Ca^2+^ channels (22) and is required for granule priming. Since its expression is reduced in T2D (40), this loss may underlie reduced Ca^2+^ channel association with granules in T2D. The association of Munc13-EGFP with granules was reduced by approximately half in human T2D versus ND β cells, in parallel with strongly reduced Ca^2+^ channel cluster density (Figure 4, A–F, M13).

### L-type channels are slowly recruited during granule priming.

**Figure 5:**
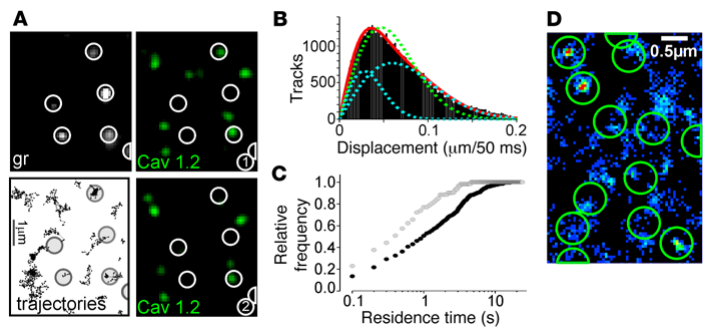
Single-molecule analysis of CaV1.2 behavior. (**A**) Single-molecule imaging of EGFP-CaV1.2 at 50 Hz. Part of an INS1 cell expressing EGFP-CaV1.2 at low levels to facilitate observation of single molecules, at 2 different time points (1–2, bandpass filtered for clarity). Granules and trajectories of individual EGFP-CaV1.2 molecules with granule positions overlaid (large circles). Scale bar: 1 μm. (**B**) Histogram of single molecule distance traveled per frame (50 ms). The red line is a fitted diffusion equation with *D_1_* = 0.007 and *D*_2_ = 0.035 μm^2^/s as diffusion coefficients; blue lines show the 2 components of the fit. The green line is the best fit, assuming a single diffusion coefficient. (**C**) Cumulative histograms of single-molecule residence times within circles of 100 nm diameter and centered at either granule (black) or random positions (gray). (**D**) Superresolution image obtained by plotting the area density of detected single molecules from a live cell. The granule positions are shown as circles. Scale bar: 0.5 μm.

We monitored the time course of EGFP-CaV1.2 and Munc13-EGFP recruitment to granules that had newly arrived at the plasma membrane (docking) in INS1 cells (Figure 4, G and H). EGFP-CaV1.2 was initially undetectable at the docking site. The ΔF/S then increased slowly and reached values similar to those at already docked granules after approximately 40 seconds (Figure 4H, green). Likewise, the ΔF/S for Munc13-EGFP only increased slowly after granule docking, although it was somewhat faster than for EGFP-CaV1.2 (Figure 4, G and H, blue). The data indicate that the 2 proteins are recruited during granule priming rather than docking. To understand the recruitment of Ca^2+^ channels to granules, we performed single-molecule imaging (Figure 5A). Single EGFP-CaV1.2 molecules, identified by step-wise bleaching and unitary brightness (Supplemental Figure 5), were mobile within the plasma membrane (Figure 3I and Supplemental Video 1). We obtained single-molecule trajectories by a tracking algorithm (41) and calculated the displacements for single-frame intervals (50 ms). A Brownian diffusion model was then fitted to the data, which revealed 2 dominant modes with diffusion coefficients of *D_1_* = 0.76 ± 0.02 and *D_2_* = 3.57 ± 0.06 × 10^–14^ m^2^/s (Figure 5B). Visually, 2 types of single-molecule behaviors were apparent: apparently random diffusion or temporary confinement to a small area, often beneath a granule (see Supplemental Video 1). On average, single-channel molecules remained for 1.06 ± 0.07 seconds within 100 nm of the granule site compared with 0.41 ± 0.06 seconds at random sites (Figure 5C). Superresolution images of EGFP-CaV1.2 constructed from live-cell, single-molecule observations (Figure 5D) indicated that EGFP-CaV1.2 molecules preferentially localized at the site of a few of the granules. Thus, CaV1.2 molecules are confined at granules but rapidly exchange with free molecules in the surrounding plasma membrane.

### Granules with associated Ca^2+^ channels undergo rapid exocytosis.

**Figure 6:**
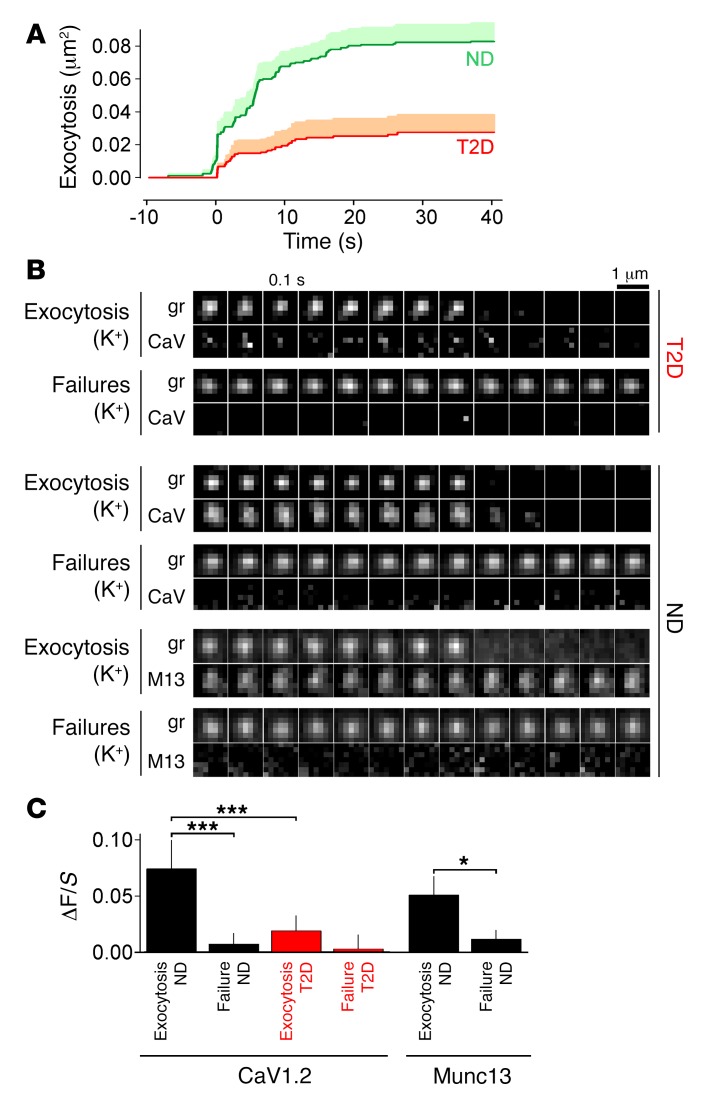
Preferential exocytosis of granules associated with L-type Ca^2+^ channels. (**A**) Cumulative time course of exocytosis in human ND or T2D cells expressing EGFP-CaV1.2 and NPY-mCherry, normalized to the cellular footprint area. Exocytosis was stimulated at *t* = 0–40 seconds with 75 mM K^+^ (ND, green, 94 events from 10 cells; T2D, red, 31 events from 12 cells; *P* < 0.0001 by Student’s *t* test). (**B**) Examples of individual granules (gr) and associated EGFP-CaV1.2 (CaV) or Munc13-EGFP (M13) signals for responder granules (Exocytosis) and failures in ND or T2D cells as indicated. Scale bar: 1 μm. (**C**) Quantitative analysis of EGFP-CaV1.2 or Munc13-EGFP binding to granules (ΔF/*S*) in **A** and **B**. *P < 0.05 and ***P < 0.001, by Student’s *t* test.

To test how association with Ca^2+^ channels affects exocytosis, we expressed EGFP-CaV1.2 and NPY-mCherry in human β cells and depolarized them with elevated K^+^ for 40 seconds. As expected, exocytosis in cells from T2D donors was only one-third of that in cells from ND donors (0.084 ± 0.06 vs. 0.027 ± 0.009 events per µm^2^, *P* < 0.001), with the strongest reduction occurring during the initial burst (Figure 6A). The corresponding EGFP-CaV1.2 signal (ΔF/S) prior to exocytosis was 4-fold stronger in ND cells than in T2D cells (Figure 6, B and C), indicating reduced L-Ca^2+^ channel association with granules in T2D. Neither exocytosis nor the location of EGFP-CaV1.2 was affected by the L-type agonist BayK8644 (5 μM, Supplemental Figure 8). However, in both NA and T2D cells, we found higher ΔF/S values at responders than at failures (Figure 6C).

**Figure 7:**
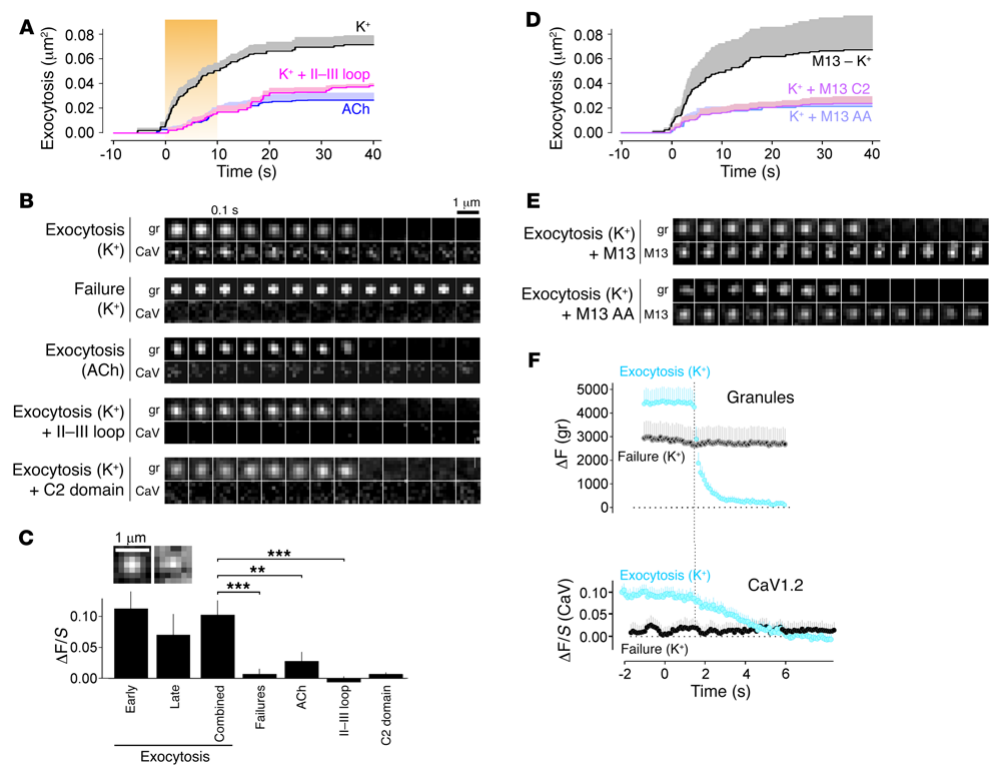
Exocytosing granules are associated with L-type Ca^2+^ channels localized to Munc13. (**A**) Cumulative time course of exocytosis in INS1 cells expressing EGFP-CaV1.2 and NPY-mCherry and stimulated with 75 mM K^+^ (black, 88 events from 13 cells) or 250 μM ACh (purple, 28 events from 9 cells) were applied at *t* = 0. Cells coexpressing the II–III loop fragment were stimulated with K^+^ (pink, 41 events from 15 cells). (**B**) Examples of individual granules and associated EGFP-CaV1.2 (CaV) signals in cells as in **A**. Scale bar: 1 μm. (**C**) Quantitative analysis of EGFP-CaV1.2 binding to granules (ΔF/*S*) in **D** and **E** for early (0–10 s, see **D**), late (10–40 s), or all responders or failures. ACh stimulation and expression of the II–III loop or Munc13 C2 domain as indicated. Images are average CaV1.2 images centered onto the granule position prior to exocytosis for early and late events. Scale bar: 1 μm. **P < 0.01 and ***P < 0.001, by Student’s *t* test. (**D** and **E**) As in **A** and **B**, but for cells expressing Munc13-EGFP (M13, gray), Munc13-AA-EGFP (M13 AA, purple) or the C2 domain fragment of Munc13 (M13 C2, pink), together with the granule marker. (**F**) Quantitative analysis of granule (upper, ΔF) and EGFP-CaV1.2 (lower, ΔF/*S*) fluorescence for the cells in **A** (black, K^+^), aligned to the moment of exocytosis for responders (Exocytosis, blue) and failures (gray); 88 granules each in 13 cells.

In INS1 cells, the depolarizations released, on average, 6.6 ± 1.4 granules (exocytosis density 0.071 ± 0.007 granules/μm^2^) (Figure 7A), and 50% of the exocytosis events occurred during the initial 5 seconds of the stimulation. This burst of exocytosis was strongly reduced or absent when the II–III loop of CaV1.2 was coexpressed to displace L-type Ca^2+^ channels from granules, or when exocytosis was elicited with ACh, to induce the release of Ca^2+^ from intracellular stores that is spatially unrelated to granules (Figure 7A). As in human cells, responder granules were associated with stronger EGFP-CaV1.2 signals than were failures (ΔF/S = 0.10 ± 0.02 versus 0.006 ± 0.001, *n* = 91 granules, 18 cells; *P* = 0.0003) (Figure 7, B and C). Consistent with a role of Ca^2+^ channel association in the initial burst of exocytosis, early responders (0–10 s) tended to have more associated EGFP-CaV1.2 than did later responders (Figure 7C), and granules with an EGFP-CaV1.2 cluster had a higher release probability than did those without (62% vs. 37% for the 45-s pulse, 91 granules). In cells overexpressing the II–III loop fragment, EGFP-CaV1.2 was no longer localized to granules, and the ΔF/S was essentially zero at both responders and failures (Figure 7, B and C). When stimulating with ACh, EGFP-CaV1.2 still localized to granules, but the ΔF/S was similar for responders and failures. We quantified these findings using a Cox regression model with an interaction term between the ΔF/S and the group (K^+^, ACh, and II–III loop). In the K^+^ group, a ΔF/S increase of 0.1 augmented the rate of exocytosis by approximately 20% (HR 1.19, 95% CI [1.08, 1.31], *P* < 0.001). In contrast, for the other 2 groups, there was no statistical evidence of an effect of the ΔF/S signal on the exocytosis rate (Figure 7C). Following exocytosis, EGFP-CaV1.2 vanished from the docking site within a few seconds of NPY-mCherry release (Figure 7F), similar to what is observed for other exocytosis-related proteins (37).

Using the same protocol, we tested the role of Munc13 in Ca^2+^ channel association with granules. Consistent with a role of Munc13 in granule priming, EGFP-Munc13 localized to responder granules but not to failures in human ND cells (Figure 6B, M13), corresponding to a more than 3-fold higher ΔF/S (Figure 6C, Munc13). Again, we turned to the use of INS1 cells for more detailed analysis. Exocytosis in cells expressing EGFP-Munc13 was similar to the control (compare Figure 7, A and D), and responder granules were associated with stronger Munc13-EGFP signals than were failures (data not shown). In contrast, exocytosis was reduced by approximately two-thirds in cells expressing either the Munc13 C2 domain or Munc13-AA-EGFP, which carries a mutation in its C2 domain that prevents Ca^2+^ channel binding (*P* < 0.001, *n* = 9 cells) (Figure 4D). Both Munc13-EGFP and Munc13-AA-EGFP localized to docked granules to a similar degree (ΔF/S = 0.08 ± 0.02, *n* = 38 cells and 0.095 ± 0.018, *n* = 35 cells, NS) (Figure 7E). The data suggest that Munc13 is involved in the recruitment of L-type channels to the release site.

### Number of L-type channels in granule-associated clusters.

The fluorescence intensities of EGFP-CaV1.2 clusters were used to estimate how many channels are present within a granule-associated cluster. The average ΔF value in the experiments depicted in Figure 4, A–F, is proportional to the copy number of EGFP-CaV1.2 molecules that are bound to the average granule site. On average, the ΔF was 97 ± 14 camera units (cu), or 1.9 ± 0.3 × 10^6^ cu/(W × s) when the exposure time (50 ms) and excitation power (1 mW) are considered. By dividing this value with the fluorescence of a single EGFP molecule (0.82 ± 0.01 × 10^6^ cu/(W × s); Supplemental Figure 5B), we derived that, on average, 2.4 ± 0.4 EGFP-CaV1.2 molecules bound to each granule. Since the ΔF is an average of all granules, but only 26% of the granules carried a channel cluster (Figure 4D), each of these granules was associated with 9.1 ± 2.1 EGFP-CaV1.2 molecules. Unlabeled endogenous L-type channels were also present and corresponded to approximately half of the whole-cell L-type current (Supplemental Figure 4, B–C). Therefore, each granule-associated cluster contained 15–20 L-type channels, which contrasts with our previous electrophysiology-based estimates of 7 channels per granule in mouse β cells (4).

## Discussion

We have identified a pool of insulin granules that is docked at the plasma membrane and associated with clusters of Ca^2+^ channels and Munc13. Upon depolarization, these granules are exposed to microdomains of high [Ca^2+^], which strongly increases their release probability and decreases their latency. As a consequence, exocytosis and insulin release are efficiently coupled to cellular electrical activity rather than the bulk cytosolic [Ca^2+^] that accumulates as a consequence of channel opening. The rapid-release kinetics and number of these granules suggest that they are identical with the immediately releasable pool (IRP) that has been defined electrophysiologically in β cells (4). T2D is associated with the loss of rapid (first-phase) insulin secretion, which we previously proposed to reflect the release of granules situated close to Ca^2+^ channels. Indeed, in T2D β cells, exocytosis was slower and not synchronized with membrane depolarizations, and neither Ca^2+^ influx nor CaV1.2 was concentrated at insulin granules. Moreover, culture in fatty acid concentrations that are diabetogenic in vivo resulted in the dissociation of Ca^2+^ channels in INS1 cells. These changes are related, as illustrated by the fact that we could induce kinetic changes similar to those in T2D cells by randomizing granule locations relative to Ca^2+^ channels (II–III loop or Munc13 C2 domain) or by randomizing the location of the Ca^2+^ source (ACh causing release from stores). The effects of Ca^2+^ channel clustering on insulin secretion will be strongest during short depolarizations, and it should be pointed out that individual glucose-dependent action potentials last only about 50 ms and their bursts no longer than a few seconds. Because of this, the lack of Ca^2+^ channel association may also underlie the disturbed first-phase release in diabetic patients. Indeed, knockout of L-type channels in mouse β cells preferentially disrupts first-phase insulin secretion (2), and HFD-induced diabetes in mice is associated with both reduced first-phase secretion and altered Ca^2+^ microdomains (42).

ACh, which releases Ca^2+^ from intracellular stores, was relatively inefficient at triggering exocytosis. This is consistent with insulin secretion measurements (43) and illustrates the importance of Ca^2+^ microdomains for efficient exocytosis. However, both the modest global cytosolic Ca^2+^ increase and the generation of diacylglycerol (DAG) in response to ACh will recruit Munc13 and related proteins such as Ca^2+^ dependent activator protein for secretion (CAPS) and double-C2 domain (Doc2) to the plasma membrane (44) and thereby accelerate granule priming (45, 46). Given our data and findings from another study (22), it can be speculated that this increase in Munc13 availability also leads to enhanced L-type channel association with granules and that both mechanisms may contribute to the rescue of first-phase secretion by ACh in diabetic GK rat islets (47).

The rate of exocytosis slowed after an initial rapid burst, which is similar to data obtained by capacitance measurements (4). Our data suggest that this slowed rate of exocytosis occurs at least in part because the Ca^2+^ channel–associated granules undergo rapid exocytosis, while their recovery by recruitment of channels onto docked granules is relatively slow. Although single L-type channels were mobile in the plasma membrane, their accumulation at the release site occurred nearly 1 minute after a granule had docked, which may be a consequence of similarly slow recruitment of the priming factor Munc13. This is consistent with the slow recovery of IRP after stimulation (4, 46, 48) and explains in part why only a fraction of the docked granules is found in this state. Thus, different release probabilities of docked granules reflect stages along a slow maturation pathway of the release site, and the copy number of Ca^2+^ channels and possibly other proteins at the release site reflects the time that has passed after docking.

We also observed exocytosis for granules situated away from Ca^2+^ channels (low ΔF/S) and between pulses when Ca^2+^ channels were closed. This may be explained by the presence of a small pool of highly Ca^2+^-sensitive granules (HCSP) with an apparent *K_D_* that is at least 10-fold lower than that of IRP granules (49). The HCSP has not yet been demonstrated in human β cells, but is suggested by a component of slow exocytosis observed in capacitance measurements (14, 20, 50). Another reason may be that channels are also present in the surrounding plasma membrane, although at lower density. The fact that some exocytosis occurred in T2D cells and in the presence of either the II–III loop or Munc13 C2 fragment suggests that even these unbound channels contribute to exocytosis, although with lower probability (Figure 3E).

Consistent with previous results (31), expression of the labeled channel did not cause increased Ca^2+^ currents. This suggests that the cells have an intrinsic mechanism to limit the number of channels on the plasma membrane. Assuming a density of 0.6 granules/μm^2^, an average INS-1 cell (700 μm^2^) contains approximately 400 docked granules, of which approximately 100 are associated with a Ca^2+^ channel cluster. On average, each of these granule-associated clusters contains 15–20 channels, or at least 1,500 in total. We acknowledge that this is higher than our earlier estimate of approximately 500 active channels in mouse β cells (4), which may reflect the larger currents observed in INS1 cells. In addition, we observed CaV1.2 away from granules, and it is unclear whether these channels are functionally equivalent to the granule-associated channels. Indeed, there is evidence that clustering on its own and coupling to granules directly affects L-type channel kinetics (30, 51). Moreover, β cells also express non–L-type channels that couple to active zones in neuronal synapses (28) and may also do so in endocrine cells.

How do granules capture the channels? L-type channel α subunits interact functionally with SNARE proteins (30, 52) and the C2 domains of synaptotagmin (23, 27) and RIM1 (23, 25, 32, 53–55), providing a structural framework for localizing the channel similar to that for neuronal synapses (21, 23, 24, 26). Neuronal Ca^2+^ channels also interact with the related C2 domain of Munc13 (22), which has not been established for L-type channels. Here, we found that expression of a C2 domain mutant or the isolated C2 domain reduced both L-type channel binding to insulin granules and rapid exocytosis, supporting the notion that L-type channels interact with the C2 domain of Munc13. We show that Munc13 is recruited only slowly to newly docked granules, which in turn could limit recruitment of L-type channels. The fact that these interactions fail in human T2D may be related to the reduced expression of soluble *N*-ethylmaleimide–sensitive factor attachment protein receptors (SNAREs) and Munc13 (56) or to their altered regulation by lipids (57) and provides a rationale for the early secretory defects associated with the disease.

## Methods

### Cells.

Human islets were dissociated and plated onto coverslips before transduction with adenovirus for expression of NPY-mCherry. INS1 cells (clone 832/13) were provided by H. Mulder (Lund University, Malmö, Sweden) and maintained as described previously (35). For experiments, cells were plated onto coverslips, transfected using Lipofectamine 2000 (Invitrogen, Thermo Fisher Scientific), and used 36–42 hours later.

### Plasmids.

The constructs used were the granule marker NPY-mCherry (38) and the same marker inserted into the second slot of the bicistronic pIRES vector [p(empty)-IRES-NPY-mCherry] (37) and the II–III loop construct pSynprint-IRES-NPY-mCherry, which was obtained by inserting a PCR fragment corresponding to amino acids 782–926 of mouse CaV1.2 using Nhe1 and EcoR1 into the first slot of p(empty)-IRES-NPY-mCherry. pLyn-rGECO had the targeting sequence of Lyn (MGCIKSKRKDG) N-terminally fused to R-GECO. To create EGFP-tagged CaV1.2, the ORF of the mouse CaV1.2 α-1C subunit isoform 3 was amplified by PCR using the corresponding IMAGE clone (Source Bioscience) as a template and cloned into the pEGFPC3 vector (Clontech). The resulting L-α-1C/pEGFPC3 construct was coding for the full-length CaV1.2 with GFP on its N-terminus separated by a 10-amino-acid peptide linker. In order to render CaV1.2 dihydropyridine (DHP) resistant, Thr 1036 was mutated to Tyr using a QuikChange XL Site-directed Mutagenesis Kit (Stratagene). A C-terminal fusion of rat Munc13.1 (NM_022861.1, NP_074052.1) with EGFP was obtained from J. Rettig (Saarland University, Saarbrücken, Germany). Amino acid residues K723 and R724 in this Munc13-EGFP were changed into alanine residues using PCR-based site-specific mutagenesis to obtain Munc13-AA-EGFP (primers: GCAGCGACAAAAACCATCTACGGGAA and CTTGGTCTTCCCAACCTGG). The cDNA region coding for the C2B domain of rat Munc13.1, amino acid residues 687–819 with the addition of a start methionine, was cloned into the x-IRES-NPY-Cherry vector using seamless PCR cloning to obtain Munc13-C2B-IRES-NPY-Cherry (primers: GGCTAGCGCCACCATGTGGTCTGCCAAAATTAGCATC, GATCTCCACACTGATGTGAAGC, and TAATAAGAATTCACGCGTCGAG).

### Solutions.

Cells were imaged in 138 mM NaCl, 5.6 mM KCl, 1.2 mM MgCl_2_, 2.6 mM CaCl_2_, 3 mM D-glucose, and 5 mM HEPES (pH 7.4 with NaOH) at 32°C, or 25°C for single-molecule imaging. For exocytosis experiments, the same buffer contained 10 mM glucose, 200 μM diazoxide, and 2 μM forskolin. Solutions containing oleate or palmitate (0.5 mM) were prepared as described previously (9). Where stated, cells were incubated in acetomethoxy (AM) esters of Fluo5F (2 μM), EGTA, or BAPTA (both at 10 μM) for 10 minutes. Exocytosis was evoked by timed local application of ACh (50 μM) or high K^+^ (75 mM equimolarly replacing Na^+^) through a pressurized glass electrode. Cells were exposed for no longer than 40 seconds, during which the effects of elevated K^+^ on cellular metabolism are likely minimal (58). We verified that the K^+^ protocol evoked rapid depolarizations to 0 mV that did not depend on action potential firing, unlike conventional stimulation with tolbutamide or 30 mM K^+^ (Supplemental Figure 6).

### Microscopy.

Cells were imaged using a custom-built lens-type TIRF microscope based on an AxioObserver Z1 with a ×100/1.45 objective (Carl Zeiss). Excitation was from 2 diode-pumped solid-state (DPSS) lasers at 491 and 561 nm (Cobolt) passed through a cleanup filter (zet405/488/561/640x; Chroma Technology) and controlled with an acousto-optical tunable filter (AA Opto Electronic). Excitation and emission light was separated using a beamsplitter (ZT405/488/561/640rpc; Chroma Technology). The emission light was chromatically separated onto separate areas of an electron-multiplying charge-coupled device (EMCCD) camera (QuantEM 512SC; Photometrics) using an image splitter (Optical Insights), with a cutoff at 565 nm (565dcxr, Chroma) and emission filters (ET525/50m and 600/50m; Chroma Technology). Scaling was 160 nm per pixel. For still images, the red and green color channels were acquired sequentially, first with cells exposed to 491 nm (1 mW) for 1 second (50 × 20 ms average), immediately followed by 561 nm (0.5 mW) for 100 ms; bleed-through from mCherry into the green channel was 0.06% ± 0.01%. For movies, cells were excited simultaneously with 491 and 561 nm light and recorded in stream mode with 100-ms exposures (10 frames/s), a 1-s exposure (1 frame/s, Figure 4, G and H), or a 50-ms exposure (Figure 5), and bleed-through was 0.6% ± 0.2%. Alignment of the red and green color channels was corrected off-line as previously described (59).

### Image analysis.

R-GECO fluorescence was corrected for out-of-cell background and measured in the entire cellular footprint (F^cell^) or in a circle of 0.5 μm (F) and divided by the prestimulation value (F^cell^_0_ or F_0_). Immobile, docked granules were identified by eye. Colocalization of EGFP-labeled proteins with granules was measured as described previously (38). Briefly, at the position of randomly selected granules (>7 per cell, well separated from other granules and the edge of the cell), we measured the average pixel green fluorescence in a) a central circle (*c*) of 3 pixels (0.5-μm) in diameter; b) a surrounding annulus (*a*) with an outer diameter of 5 pixels (0.8-μm); and c) a background area not touching any cell (*bg*). The circle contains all of the fluorescence originating from the docking site; it also contains fluorescence from molecules not bound to the docking site, which is estimated using *a*. To obtain the specific on-granule fluorescence ΔF, the annulus value (*a*) was therefore subtracted from that of the circle (*c*) (ΔF = *c* – *a*). To obtain off-granule fluorescence, the annulus value was background corrected (*S* = *a* – *bg*). *S* represents the local unbound concentration of the labeled protein, and averaged for each cell, *S* is linearly related to the protein’s expression level. For many proteins, the relationship of ΔF to *S* follows a 1-site binding equation that reaches saturation at higher expression levels (37, 60). For a relatively small *S*, the ratio of ΔF/S is a convenient measure of protein binding to the docking site, which is independent of the expression level. Positive ΔF/S values indicate binding to the docking site, and negative values indicate exclusion. Note that the latter can occur for proteins with cytosolic expression due to exclusion by the granule volume. For untargeted EGFP, we found ΔF/S = –0.06. Colocalization was also estimated by an observer; a computer presented square cutouts of the green channel centered on the position of the granules, allowing the user to decide whether a cluster was present or not. Granule density was calculated using the “find maxima” function in ImageJ (NIH; http://rsbweb.nih.gov/ij). Exocytosis, docking and visiting events were detected manually (37, 61); exocytosis events had signal/noise ratios of approximately 5, were completed in less than 1 second, and were easily distinguished from rare undocking events. Rise times (*t_1/2_*) at each granule were obtained by fitting a Hill expression F = F_max_ *t*^n^/[(*t_1/2_*)^n^ + *t*^n^] to the signal during the first K^+^ pulse. Single molecules were traced using the ImageJ plug-in Particle Tracker (41) or ImageJ QuickPALM (62).

### Mathematical modeling.

The spatiotemporal profile of calcium concentrations was simulated by solving partial differential equations (PDEs) describing diffusion and mutual binding of Ca^2+^ ions and buffer molecules. The realization of stochastic channel gating was used to calculate Ca^2+^ influx as input for the PDE model. The cell was represented by a sphere (radius = 6.5 μm), and all Ca^2+^ channel clusters (3–20 channels per cluster) were equivalent and uniformly distributed over its surface. The low density of Ca^2+^ channels in β cells (4) allowed us to restrict the simulations to a conical region with a base radius of 1.5 μm, with the Ca^2+^ current source located at the base center of the conical region. We assumed no flux boundary conditions for Ca^2+^ and buffers on the sides of the cone, assuming that Ca^2+^ and buffer fluxes flowing into the cone from the neighboring regions are balanced by equal reverse fluxes. Because of the conical geometry, the full 3D problem was reduced to a 2D problem, using rotationally symmetric spherical coordinates, thus reducing the computational intensity. The Ca^2+^ simulations included 3 types of buffers. The membrane-bound R-GECO sensor was assumed to be immobile and confined to a thin layer under the cone base. A total concentration (*C_T_*) of 20 μM and a thickness of 50 nm for the layer in which the buffer is present were assumed, corresponding to approximately 600 molecules/μm^2^. Kinetics, rate constants, and affinity for R-GECO were taken from the literature (63). The second buffer was EGTA (none or 1 mM) with characteristics as previously described (64). Finally, a generic endogenous buffer (both mobile and immobile) was included. The single Ca^2+^ channel current is *i*_Ca_ = *g*_Ca_ (*V – V*_Ca_), where the *g*_Ca_ of approximately 2 picosiemens is the single-channel conductance (65) and the calcium reversal potential *V*_Ca_ is approximately 65 mV (in 2.6 mM extracellular Ca^2+^). Depolarizing the cell with 75 mM K^+^ results in a membrane potential (*V*) of approximately 0 mV (Supplemental Figure 6) (66), giving a single-channel current of approximately 0.13 pA. The reaction-diffusion equations for Ca^2+^ and buffers were solved using Calcium Calculator (CalC) software (http://www.calciumcalculator.org) (67). CalC uses an alternating-direction implicit finite difference method, which is second-order accurate in spatial and temporal resolution, and an adaptive time-step method. We used a nonuniform spatial grid with a stretch factor of 1.03. The simulated, spatiotemporal Ca^2+^-bound GECO levels were post-processed by convolving with the point spread function (PSF) of the microscope and averaged over the acquisition time (100 ms). MATLAB (MathWorks) was used to simulate channel gating and to perform post-processing. The simulations of Ca^2+^ influx quantitatively support the conclusion that Ca^2+^ influx occurred near granules. However, the spatial Ca^2+^ gradients that develop at the channel pore are blurred by limitations of the indicator and the microscope. Instead, we used the rise time of the local Ca^2+^ signal to estimate distances of granules from the nearest channel cluster. Simulations showed that the rise time, in contrast to the signal amplitude, is nearly independent of the number of channels per cluster. Measured rise times are limited by the finite speed of the K^+^-mediated depolarization (~50 ms, Supplemental Figure 6) and the frame rate (100 ms), and the lowest derived distances are therefore likely to be overestimated.

### Statistics.

Data are presented as the mean ± SEM unless otherwise stated. Statistical significance was assessed using Students *t* test for 2-tailed, paired or unpaired samples, as appropriate. A *P* value of less than 0.05 was considered statistically significant. To test whether exocytosis was more frequent during defined time periods (K^+^ pulses) and whether there were differences between healthy and diabetic cells, we used χ^2^ tests (for INS1 cells) and a logistic regression model (for human cells), adjusting for the different durations of K^+^ pulses and intervals between pulses. We determined the rise time of the experimental R-GECO signal (*t_1/2_*) at each granule by fitting a Hill expression F = F_max_ *t*^n^/[(*t_1/2_*)^n^ + *t*^n^] to the R-GECO signal during the first pulse, granule by granule. The ΔF/S signal for EGFP-CaV1.2 was calculated as the average over the 10 seconds before the stimulus. To quantify how *t_1/2_* influences the rate of exocytosis, we fitted Cox’s proportional hazards regression models with, respectively, log(*t_1/2_*) or the ΔF/S signal as a covariate. We tested for evidence for a potential time-varying effect of the rise time on the rate of exocytosis in the data, but found that the data were well described by a time-constant effect of rise time or ΔF/S, respectively. To account for cell-to-cell variation, granules within a cell were considered clustered data, and a marginal Cox model was used to obtain valid estimates of standard errors (36). To relate *t_1/2_* to the granule-channel distance, we fitted the rise in the simulated, processed R-GECO signal at various distances to a Hill expression, as done for the experimental data. The relation allowed us to go from distance to rise time, and with the results from the Cox regression model, to the rate of exocytosis. Statistical analysis was done in R (www.r-project.org) and the fitting for Figure 7 in Origin (OriginLab).

### Study approval.

Human pancreatic islets were isolated and provided by the Nordic Network for Clinical Islet Transplantation (Uppsala, Sweden) with full ethics board approval and informed consent (for donor information, see Supplemental Figure 9). The study was approved by the Uppsala Regional Ethics Board (2006/348).

## Author contributions

NRG, PY, and SB performed and analyzed imaging experiments. PY and PEL performed electrophysiology experiments. SB and NRG designed experiments. MR and MGP performed modeling and analyzed data, MGP conceived modeling, and VM and AS consulted the modeling. MVC and PR generated EGFP-CaV1.2. GC conceived and performed the statistical analyses. SB conceived the study and wrote the manuscript. All authors gave feedback and approved the final version of the manuscript.

## Supplementary Material

Supplemental data

Supplemental Video 1
