# Supplementary material for: Ca2+ channel clustering with insulin-containing granules is disturbed in type 2 diabetes
Source: J Clin Invest. 2017 May 8;127(6):2353–64. doi: 10.1172/JCI88491 (PMC5451232; doi:10.1172/JCI88491)
Supplement: Supplemental data [file jci-127-88491-s001.pdf]

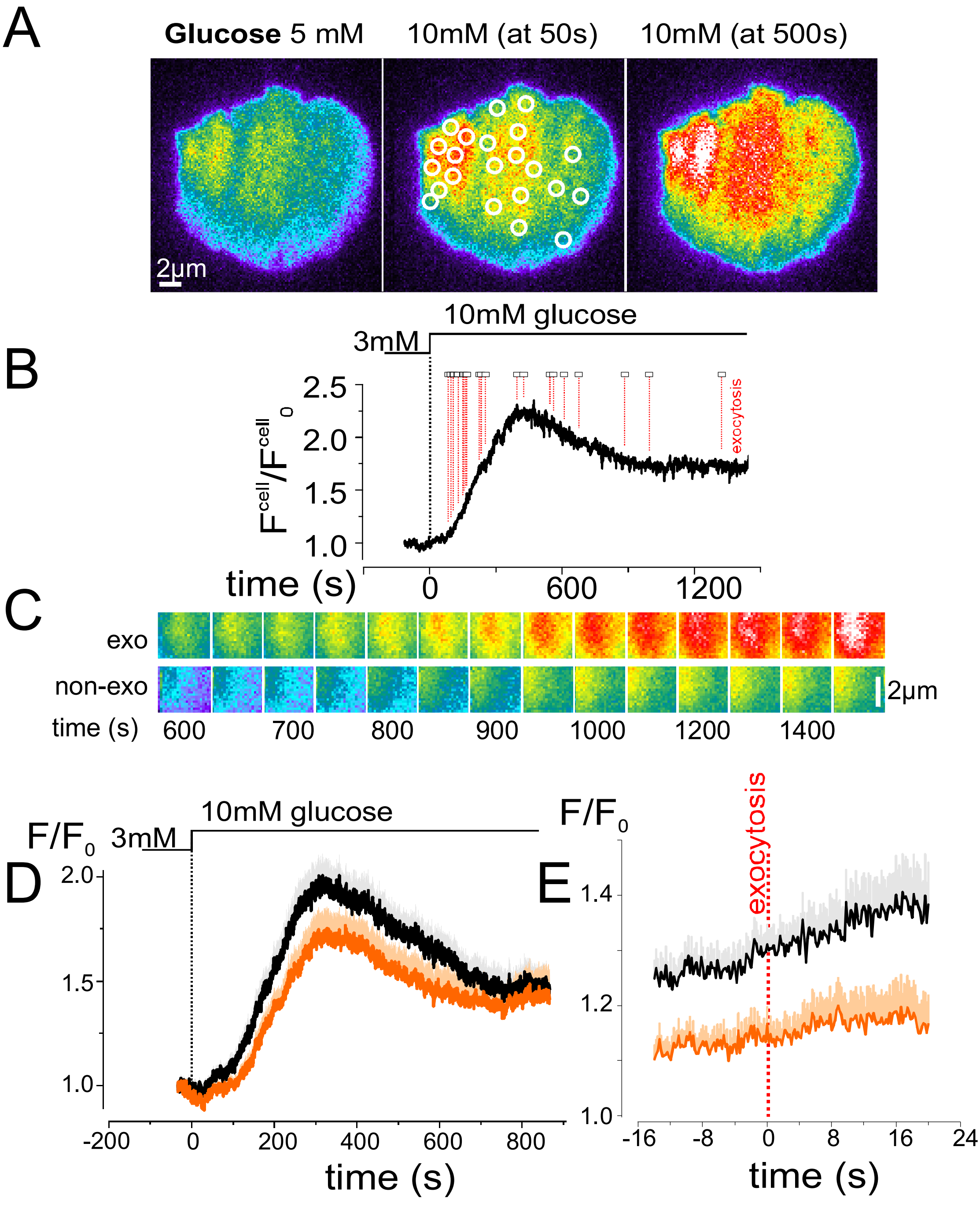


# Figure S1

**Figure S1 Granule localized $Ca^{2+}$-influx in glucose stimulated human β-cells**

**A** TIRF images of a human β-cells loaded with Fluo5F (no EGTA) before and during exposure to elevated glucose at times indicated. White circles indicate the location of granules that underwent exocytosis (Based on expression of NPY-cherry, images not shown).

**B** Cellular Fluo5F signal for the cell in A, corrected for out-of-call background and normalized to pre-stimulatory value. The time of exocytosis events is indicated by vertical orange lines.

**C** Spatially aligned Fluo5F image sequence centered at the location of all exocytosis granules (exo, upper) or random locations (non-exo, lower), at times relative to the onset of 10mM glucose as indicated. Note fluorescence increase that is centered on the position of exocytosis. N=26 exocytosis events in 5 cells.

**D** Average Fluo5F signal at all granules undergoing exocytosis (black) and at random locations (red) in the same cells.

**E** As in D, but aligned to the moment of exocytosis.

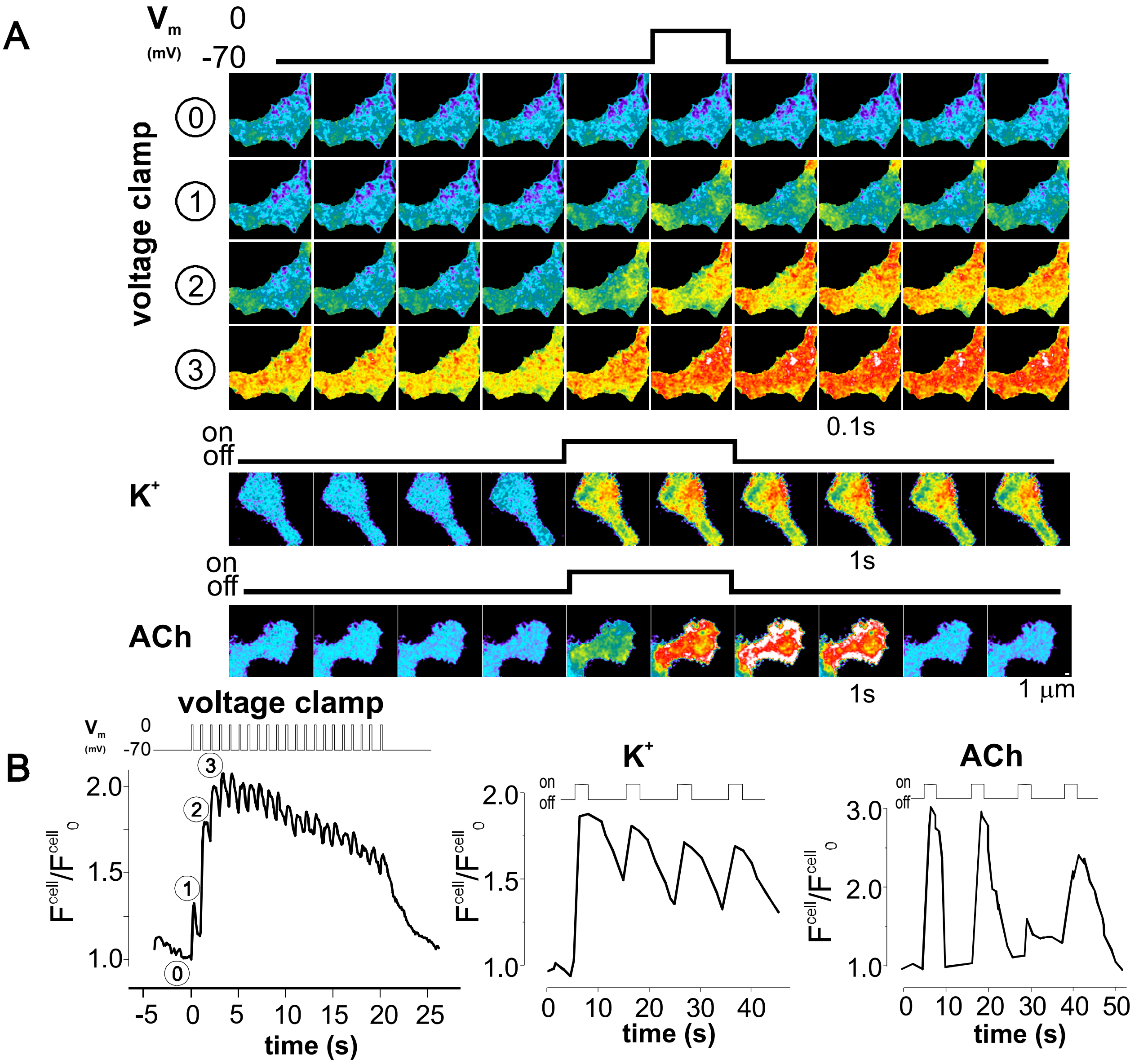


# Figure S2

**Figure S2 lyn-GECO detects local $Ca^{2+}$-signaling**

**A** Image sequences of Lyn-R-GECO fluorescence in an INS1-cell stimulated by voltage-clamp depolarizations (200 ms, 1Hz), elevated $K^+$ or acetylcholine (ACh). Three consecutive depolarizations (1-3) are shown for the voltage clamp experiment.

**B** Time course of the normalized whole cell fluorescence for the cells in A.

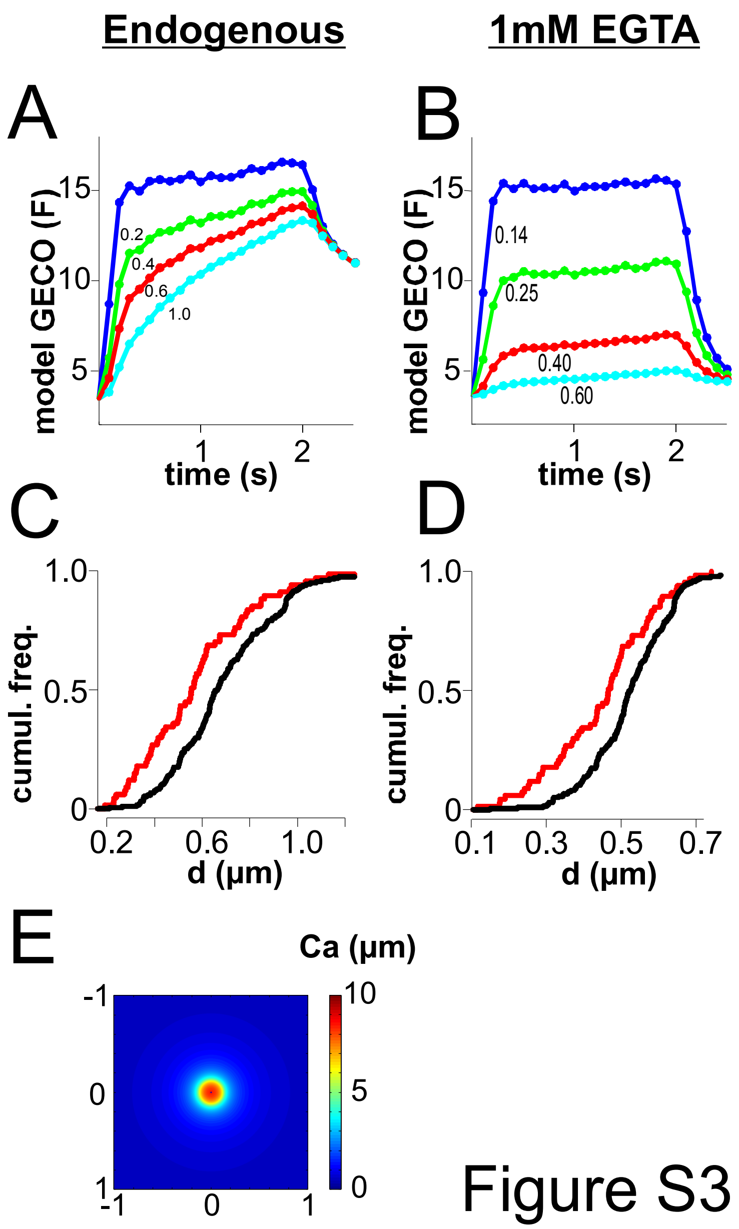


Figure S3

**Figure S3 Modeling of $Ca^{2+}$-influx**

**A-B** Modeled GECO/$Ca^{2+}$ signal assuming endogenous buffering (A) or 1 mM EGTA (B), from a cluster of 15 $Ca^{2+}$ channels and at different distances (as indicated, in µm).

**C-D** Cumulative histograms as in Fig 2K, but using the distances derived from modeling of $Ca^{2+}$influx, for responders (red) and failures (black). C, endogenous buffering, D, 1 mM EGTA.

**E** Simulated [$Ca^{2+}$] vs distance plot during $Ca^{2+}$ influx through a cluster of 15 channels, endogenous buffering. *Cf* gray trace in Fig 2L.

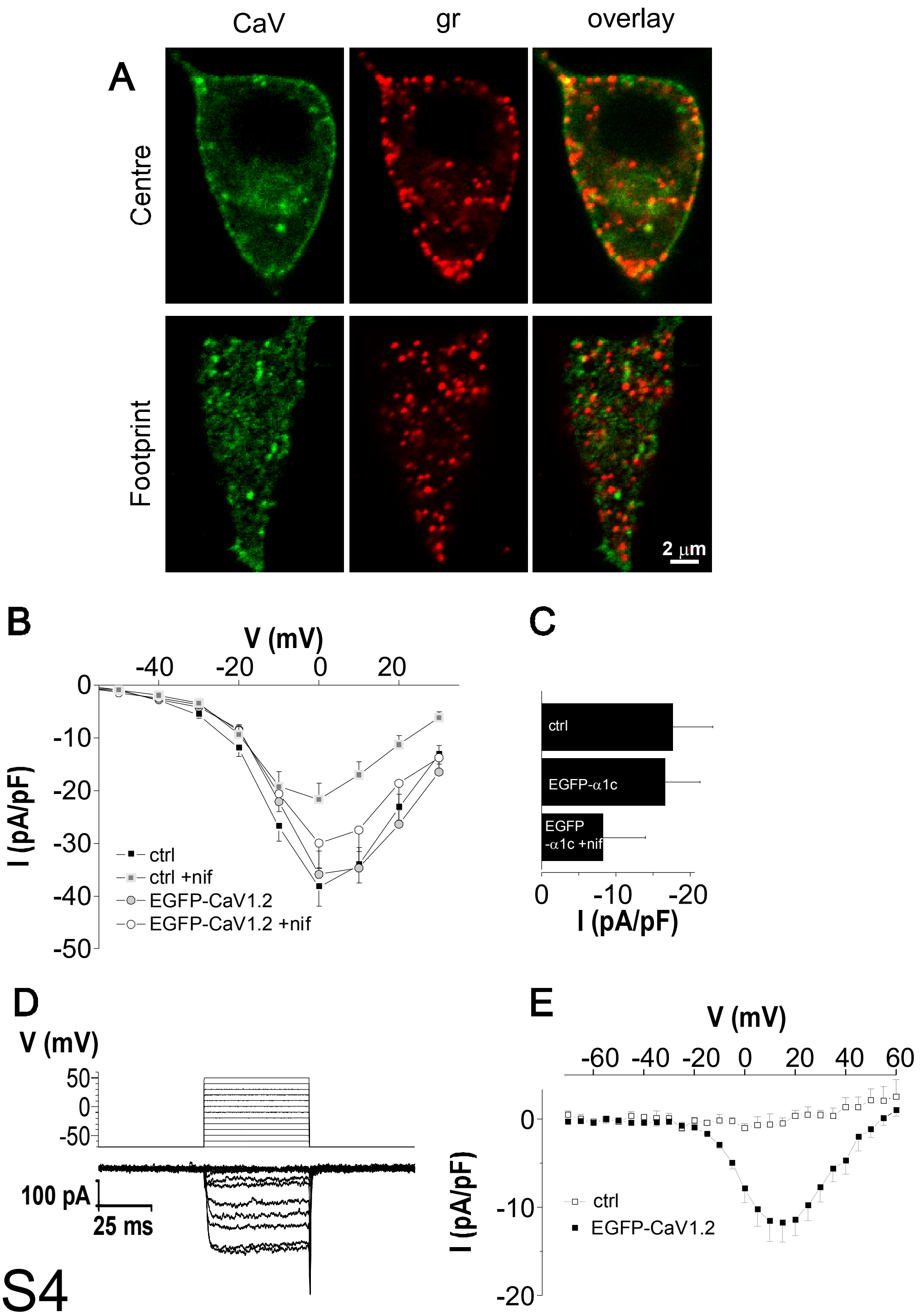


# Figure S4

**Figure S4 EGFP-CaV1.2 forms functional channels that partially replace endogenous CaV1.2**

**A** Confocal images of EGFP-CaV1.2 (green) and granule marker (red) expressed in an INS1 cell. Fluorescence from selected granules is plotted as *ΔF/S* for calcium channels at granule locations. The same cell is shown focused at the center (upper) or at the plasma membrane touching the coverslip (lower).

**B** Current-voltage (*I-V*) relationships in INS1 cells expressing EGFP-CaV1.2 with $Ba^{2+}$ as charge carrier. Cells were depolarized for 200 ms from -70mV to the indicated voltages (*V*), and average currents calculated and normalized for cell capacitance (*I*); n=15-23 cells each. Note that the L-type blocker nifedipine (nif) reduced the maximum current in non-transfected cells by 44%. In transfected cells, the effect was limited (-17%), because the expressed EGFP-CaV1.2 channels carried mutations rendering them insensitive to dihydropyridines . Half-maximal activation in the absence of nifedipine was similar in transfected and untransfected cells (-7.2±0.6, n=19 vs -8.5±1.3 mV, n=17).

**C** Current responses to zero mV depolarizations for conditions as in C. Non-L-type currents, measured in untransfected control cells in presence of nifedipine, were subtracted. Errors (SEM) are calculated using standard methods for error propagation. Note that the currents in untransfected cells with nifedipine corresponds to endogenous non-L-type $Ca^{2+}$-channels; by subtracting this current we isolated the L-type component. The data indicate that L-type currents were similar in transfected and untransfected cells, suggesting that the endogenous L-type current is partially suppressed by EGFP-CaV1.2, similar to what has been reported for a C-terminally tagged channel CaV1.2-EGFP . In transfected cells, about half of the L-type component was nifedipine-sensitive, indicating that the labeled, nifedipine insensitive channel was functionally expressed at similar or slightly lower levels than endogenous L-channels.

**D** Voltage-clamp protocol (upper) and current responses in a HEK cell transfected with EGFP-CaV1.2.

**E** Current-Voltage relationship (±SEM) in HEK cells transfected with EGFP-CaV1.2 (filled symbols) or untransfected (open symbols). n=4 and 5 cells.

In B-E the intracellular solution consisted of (in mM) 125 CsCl, 1 $MgCl_2$, 3 ATP-Mg, 0.1 cAMP, 10 EGTA, and 10 HEPES (pH 7.15). EC 128 NaCl, 5.6 KCl, 1.2 $MgCl_2$, 10 $BaCl_2$, and 10 HEPES, (pH 7.4 using NaOH)

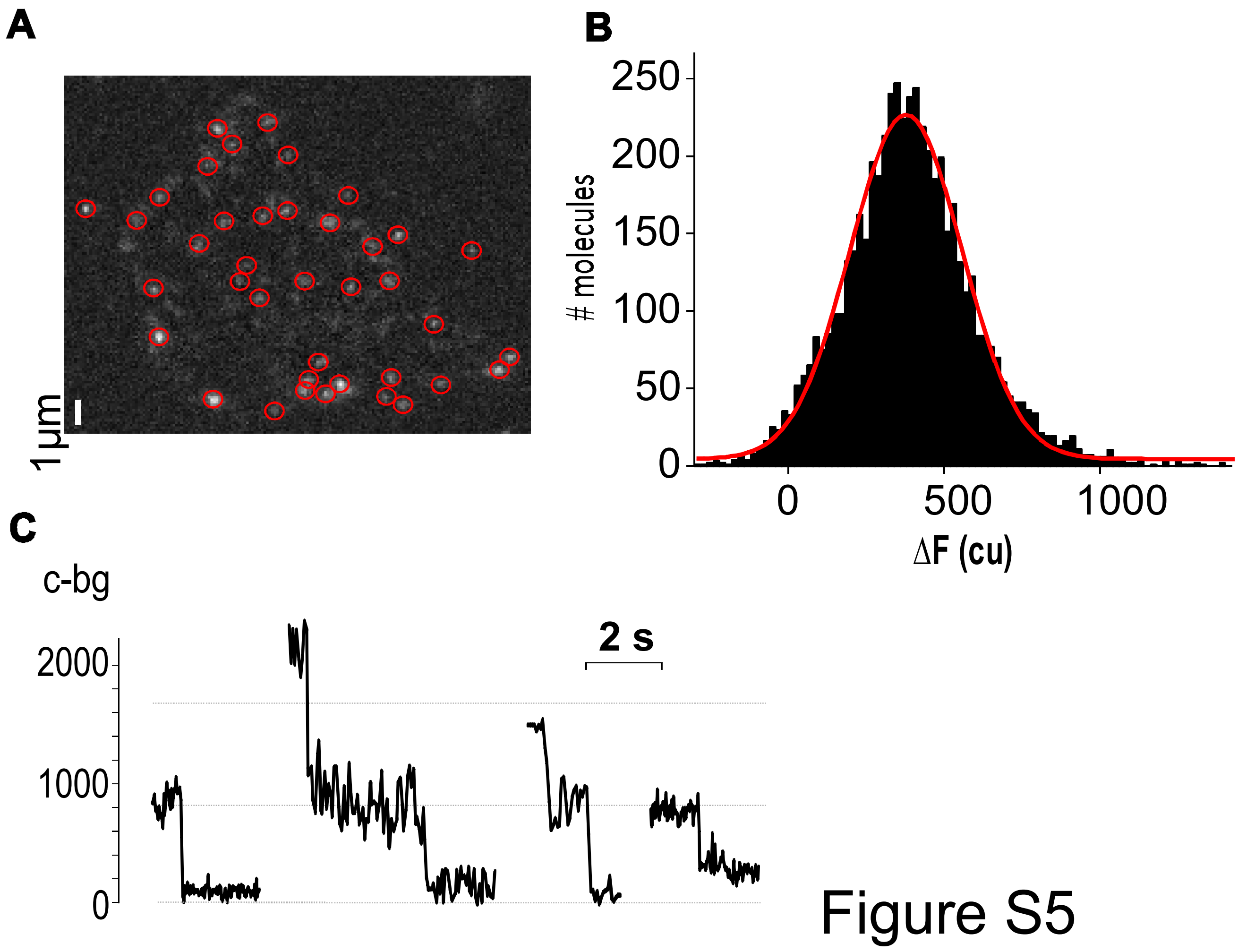


**Figure S5. Determination of single EGFP molecule fluorescence.**

**A** Image of a cell expressing a low density of EGFP-Cav1.2 molecules. Fluorescence spots were automatically identified (findspots, MetaMorph), and the fluorescence at these sites measured as *ΔF*.

**B** Histogram of the *ΔF* values in cells as in A. The peak of the overlaid Gaussian is at *ΔF*=408 *cu* (n=5 cells, 8203 spots). Since the images were recorded with 10 mW and 50 ms long exposures, the peak value corresponds to $0.816 \pm 0.01 * 10^6$ $cu * W^{-1} * s^{-1}$ and represents the fluorescence of a single EGFP molecule in our system.

**C** Four examples of step-wise bleaching (average fluorescence in a 3 pxl wide circle, subtracted for out-of-cell background; *c-bg*). In one of the examples more than one molecule was initially present, of which the last bleached 7 s after onset of the recording.

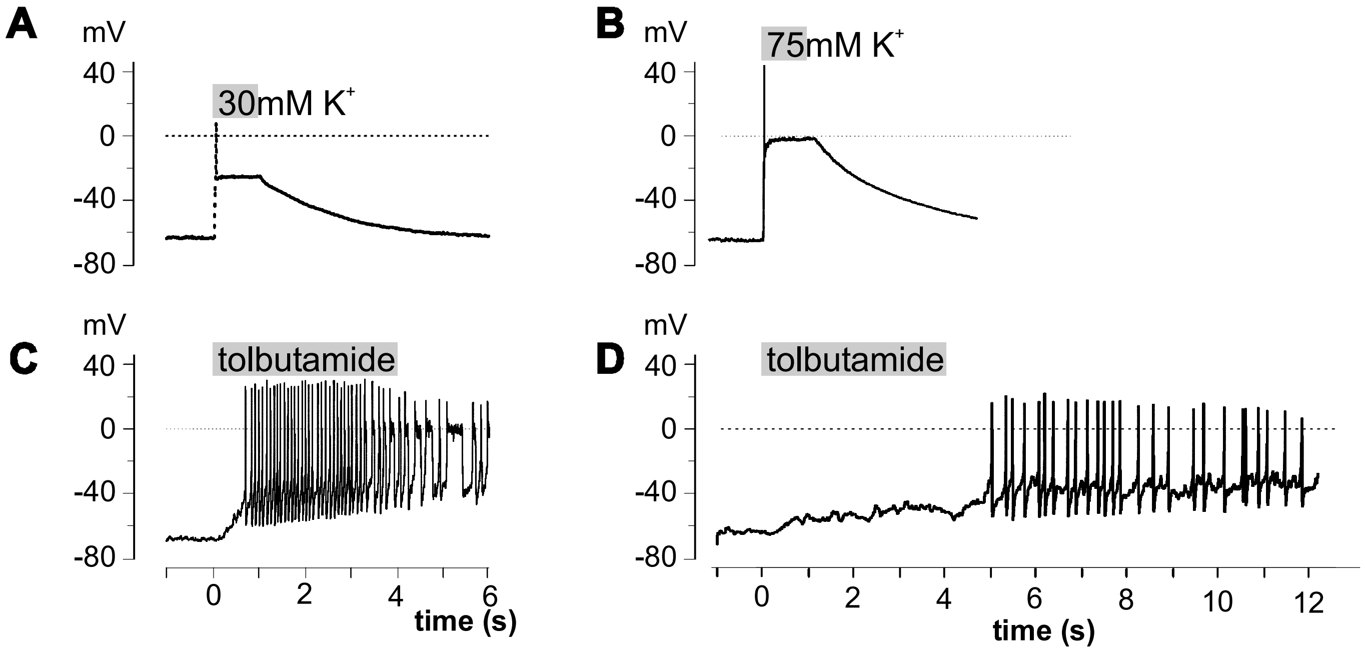


# Figure S6

**Figure S6. Characterization of computer controlled single cell application of K+ and tolbutamide**
**A-D** Representative current-clamp measurements, of the membrane potential of INS1 cells during pressure application of $K^+$ or tolbutamide.in presence of diazoxide. Elevated $K^+$ (**A** 30 mM, n=7 cells; and **B** 75 mM, n=10 cells) or tolbutamide (**C-D**, 100 µM, n=3 cells) were applied to the cells during the indicated time intervals using a glass micro pipette, identical to those used for patch clamp and air-pressurized using a computer controlled valve. Time-to-peak and plateau values were 59±3.2 ms and -24±2.5 mV in A, 43.3±1.7 ms and -6±1.2 mV in B, n.d. in C and D. Solutions were, pipette (in mM): 76 $K_2SO_4$, 10 KCl, 10 NaCl, 1 $MgCl_2$ and 5 HEPES, (pH 7.2 using KOH), Amphotericin B 240 µg/ml; and bath 138 NaCl, 5.6 KCl, 2.6 $CaCl_2$, 1.2 $MgCl_2$ 5 HEPES and 3 Glucose (pH 7.4 using NaOH)

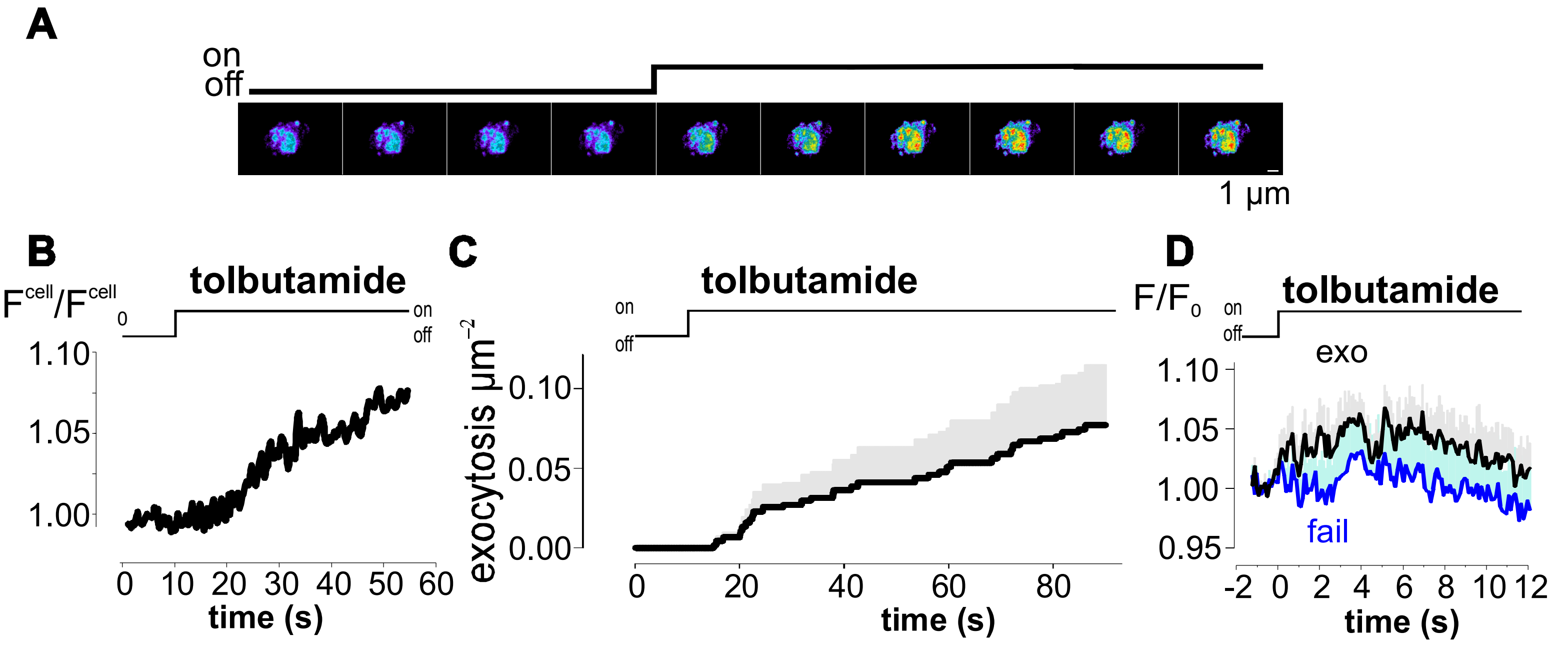


# Figure S7

**Figure S7. Calcium influx and exocytosis evoked by addition of tolbutamide.**

**A** Image sequence of Fluo5F fluorescence in a human ND β-cell stimulated with 500 µM tolbutamide (no diazoxide, 3 mM glucose). Images are separated by 5 s.

**B** Time course of the normalized whole cell Fluo5F fluorescence ($F^{cell}/F^{cell}_0$) in A. Note the slow onset after stimulations, in agreement with Fig S6D.

**C** Cumulative exocytosis events measured by imaging NPY-mCherry in 7 cells as in A; tolbutamide was applied from t=10 s.

**D** Average local Fluo5F-fluorescence at granules ($F/F_0$) undergoing exocytosis (black) or failures (blue) in response to the tolbutamide stimulation for cells that responded to tolbutamide within 2 s (6 cells, 20 granules from 2 donors).

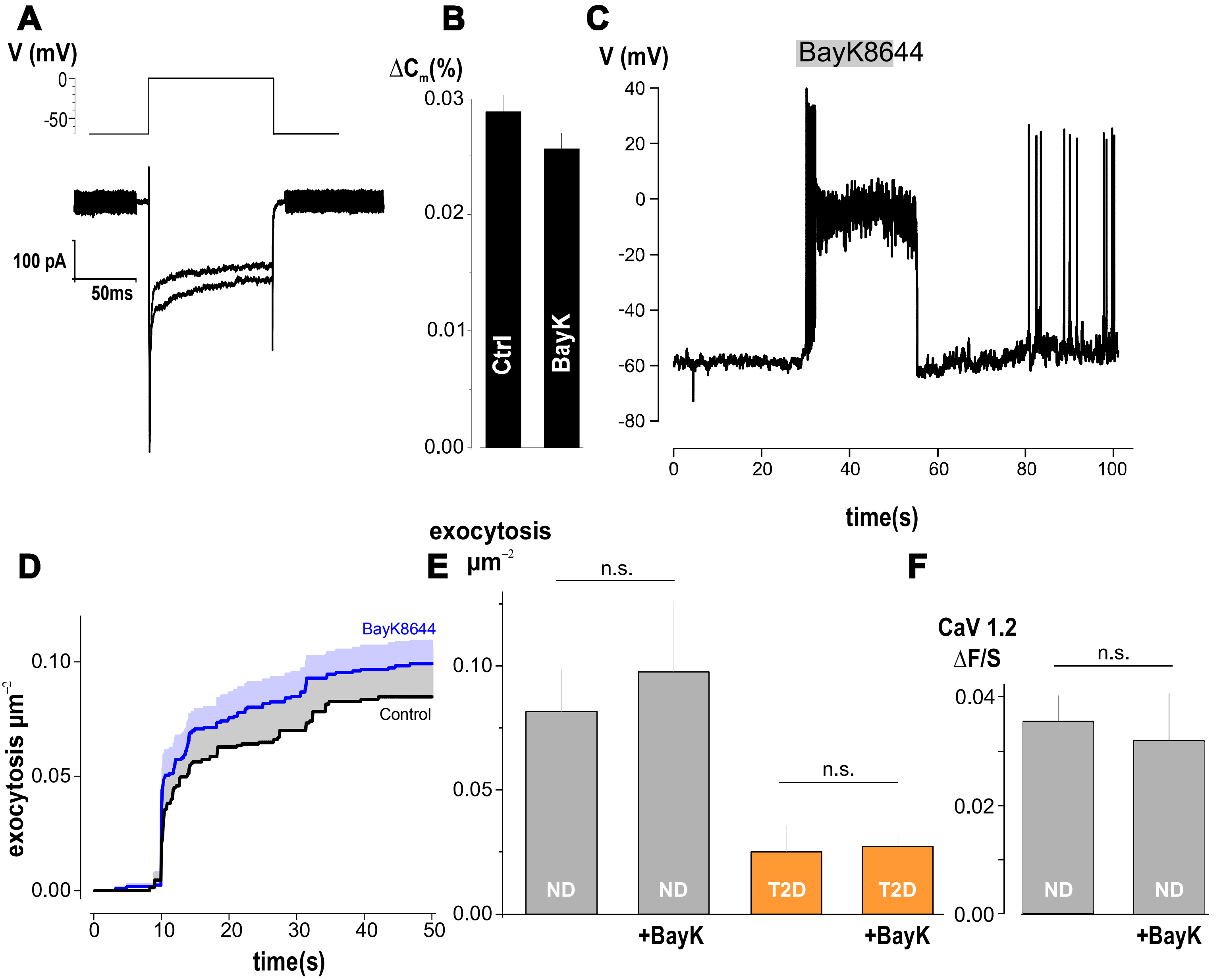


# Figure S8

**Figure S8. BayK8644 increases $Ca^{2+}$-currents and depolarizes, but has no direct effect on exocytosis.**

**A** $Ca^{2+}$ currents in presence or absence of 5 µM BayK8644 in response to voltage-clamp depolarizations from -70 to 0 mV in Ins1 cells. With BayK8644 the currents increased on average 17±3 % (n=6 cells). Solutions were(in mM): pipette, 125 Cs-glutamate, 10 CsCl, 10 NaCl, 2 $CaCl_2$, 1 $MgCl_2$, 5 EGTA, 3 Mg-ATP, 0.1 cAMP, 5 HEPES (pH 7.2 using CsOH) and bath, 138 NaCl, 5.6 KCl, 2.6 $CaCl_2$ 1.2 $MgCl_2$,5 HEPES and 3 Glucose (pH 7.4 using NaOH).

**B** Quantification of depolarization-induced exocytosis measured as capacitance increase in 6 experiments as in A.

**C** Current-clamp measurement of membrane potential in an Ins1 cells, in the absence of diazoxide. BayK8644 (5 µM) was applied to the cell during the indicated time interval. Note the long-lasting depolarization evoked by BayK8644. Solutions were, pipette (in mM): 76 K2SO4, 10 KCl, 10 NaCl, 1 $MgCl_2$ and 5 HEPES, (pH 7.2 using KOH), Amphotericin B 240 µg/ml. and bath 138 NaCl, 5.6 KCl, 2.6 $CaCl_2$, 1.2 $MgCl_2$, 5 HEPES and 3 Glucose (pH 7.4 using NaOH).

**D** Cumulative exocytosis measured by imaging NPY-mCherry in ND human β-cells; exocytosis was stimulated at t=10 s with 75 mM $K^+$ in presence of diazoxide and 5 µM BayK8644 (85 granules from 10 cells) or control (62 granules from 9 cells).

**E** Sum of exocytosis in experiments as in E, in cells from T2D (8 cells, 2 donors) or ND donors (10 cells, 3 donors).

**F** Quantification of EGFP-CaV1.2 association with granules (*ΔF/S*) in ND human cells, in presence (34 cells, 3 donors) or absence of BayK8644 (24 cells, 2 donors).

| | Donor ID | Gender | Age | BMI | HbA1c |
|---|---|---|---|---|---|
| ND | 1993 | Female | 43 | 24.9 | 5.6 |
| | 2016 | Male | 62 | 33.2 | 5.7 |
| | 2081 | Male | 19 | 21 | 5.3 |
| | 2150 | Male | 60 | 27.8 | 5.3 |
| | 2152 | Female | 57 | 34.1 | 5.9 |
| | 2155 | Male | 67 | 26.1 | 5.6 |
| | 2176 | Female | 64 | 25.7 | 5.6 |
| | 2188 | Female | 57 | 19.4 | 5.7 |
| T2D | 2202 | Female | 59 | 29.4 | 6.6 |
| | 2203 | Male | 59 | 50.9 | 6.3 |
| | 2211 | Female | 63 | 37 | 7.9 |
| | 2219 | Male | 61 | 37 | 6.1 |
| | 2121 | Male | 65 | 29.1 | 6.1 |
| | 2123 | Male | 68 | 29.3 | 6.1 |
| | 2199 | Male | 59 | 26.9 | 6.3 |

Figure S9 (Table)

**Figure S9.** (Table) showing human islet donor information.
